# The Bacilli of Tubercle

**Published:** 1882-10

**Authors:** 


THE BACILLI OF TUBERCLE.—For the demonstration of tubercle bacilli in the sputum of phthisical patients, Baumgarten recommends the following method as more convenient than those of Koch and Ehrlich. A little of the sputum is dried on the cover-glass, as recommended by the latter, and then treated with potash —one or two drops of a thirty-three per cent. solution of caustic potash added to a watch-glass of distilled water. The tubercle bacilli can then be readily seen with a magnifying power of four hundred or five hundred diameters, and a little pressure renders them still more distinct from the inclosing detritus of tissue. In order to preclude the possibility of coufounding the bacilli of tubercle with those of other species, the cover-glass may be raised and placed aside until the layer of fluid on its under surface is dry, and then passed two or three times through a gas-flame, and then on it may be placed a drop of an ordinary watery solution of aniline violet, or any other nucleus-tinting preparation of aniline. All the putrefaction bacteria then appear under the microscope as an intense blue or brown (according to the testing agent and its strength), while the tubercle bacilli remain absolutely colorless, and can be seen with the same distinctness as in the ordinary potash preparation. The whole process does not occupy more than ten minutes.—*The Lancet.*

